# Trends in Antihyperglycemic Medication Prescriptions and Hypoglycemia in Older Adults: 2002-2013

**DOI:** 10.1371/journal.pone.0137596

**Published:** 2015-09-03

**Authors:** Kristin K. Clemens, Salimah Shariff, Kuan Liu, Irene Hramiak, Jeffrey L. Mahon, Eric McArthur, Amit X. Garg

**Affiliations:** 1 Department of Medicine, Western University, London, Ontario, Canada; 2 Institute for Clinical Evaluative Sciences, Ontario, Canada; 3 Department of Medicine, Division of Endocrinology, Western University, London, Ontario, Canada; 4 Department of Epidemiology and Biostatistics, Western University, London, Ontario, Canada; University of Catanzaro Magna Graecia, ITALY

## Abstract

**Background:**

Over the last decade, several new antihyperglycemic medications have been introduced including those associated with a lower hypoglycemia risk. We aimed to investigate how these medications are being prescribed to older adults in our region.

**Methods:**

We conducted population-based cross-sectional analyses of older adults (mean age 75 years) with treated diabetes in Ontario, Canada from 2002 until 2013, to examine the percentage prescribed insulin, sulphonylureas, alpha-glucosidase inhibitors, metformin, thiazolidinediones, meglitinides, and dipeptidyl peptidase-4 inhibitors. Over the study period, we also examined their hospital encounters for hypoglycemia (emergency room or inpatient encounter).

**Results:**

The mean age of treated patients increased slightly over the study quarters and the proportion that were women declined. With the exception of chronic kidney disease, cancer, dementia, and neuropathy, the percentage with a comorbidity appeared to decline. The percentage of treated patients prescribed metformin, gliclazide and dipeptidyl peptidase-4 inhibitors increased as did combination therapy. Glyburide and thiazolidinedione prescriptions declined, and insulin use remained stable. In those with newly treated diabetes, the majority were prescribed metformin, with smaller percentages prescribed insulin and other oral agents. Although the absolute number of treated patients with a hypoglycemia encounter increased until mid-2006 and then decreased, the overall percentage with an encounter declined over the study period (0.8% with an event in the first quarter, 0.4% with an event in the last quarter).

**Conclusions:**

Antihyperglycemic medications with safer profiles are being increasingly prescribed to older adults. In this setting there has been a decrease in the percentage of treated patients with a hospital encounter for hypoglycemia.

## Introduction

The management of glycemic control in older patients with diabetes has become increasingly complex over the last decade. [1] First, where only sulfonylureas (eg. glyburide), insulin, alpha glucosidase inhibitors (eg. acarbose), and biguanides (eg. metformin) were accessible in Canada in the 1990’s, there are now 9 classes of medications and at least 20 unique drugs and their combinations available to control hyperglycemia. Second, while all drugs by design lower glucose levels, there are important differences among them with respect to their other known or suspected advantages and risks. Of particular importance in older patients are differences among the medications in risk for hypoglycemia. [2–4] Third, while randomized trials have established the benefit of intensified glycemic control in reducing the risk for microvascular complications, it remains unclear as to whether this also leads to an important reduction in the risk for macrovascular complications and, if so, whether such benefit exceeds the risks of tighter control in all cases. [5,6]

Given there are limited data on how antihyperglycemic medications are being prescribed to older patients with diabetes, in the current study we aimed to examine the prescription trends of these medications in this population from 2002 until 2013 in our region (Ontario, Canada). As the hypoglycemia risk of these medications differ, we also examined hospital encounters for hypoglycemia amongst treated patients over the period of study.

## Materials and Methods

We conducted population-based cross sectional analyses of older adults with diabetes in Ontario, Canada from April 1, 2002 until March 31, 2013, using linked health care databases. Ontario currently has a population of over 13 million people, of which 2 million are age 65 years or older. [7] In our province, people over the age of 65 have universal coverage for outpatient prescription medications, physician services, hospitalizations and investigations. [8]

We divided our study timeframe into 3-month intervals (study quarters). We report this study using guidelines for observational studies (checklist of recommendations presented in S1 Table). [9]

### Ethics Statement

The databases were linked together using unique encoded identifiers that enable complete and accurate linkage of patient records across the databases. These encoded identifiers allowed patient records to be anonymized and de-identified prior to analysis. Data analysis took place at the Institute for Clinical Evaluative Sciences (ICES) according to a pre-specified protocol. The study was approved by the research ethics board at Sunnybrook Health Sciences Centre (Toronto, Canada). Informed consent was not required from patients, as ICES is a named entity under Ontario’s Personal Health Information Protection Act and is able to receive and use health information without consent in order to examine the province’s health care system.

### Data Sources

We used 6 databases to examine patient characteristics, drug use, covariate information, and outcomes. To identify patients with diabetes, we used the Ontario Diabetes Database (ODD). This database captures patients with any diagnosis of diabetes (eg. type 1 diabetes, type 2 diabetes) based upon 2 physician service claims for diabetes in the 2 years previous or 1 hospitalization with a diagnostic code for diabetes. [10] Women with gestational diabetes are excluded from this database. The ODD has been described in detail previously and has been found to have an 86% sensitivity and 97% specificity to detect diabetes. [10] The Registered Persons Database of Ontario was used to collect vital statistics. It contains demographic information on all Ontario residents who have ever been issued a health card. We used the Ontario Drug Benefit Program database to examine prescription medications as in our province, adults age 65 and older are eligible for drug coverage, and the information on these prescribed medications is accurately contained within this database (error rate of less than 1%). [11] Diagnostic and procedural information on hospitalizations and emergency room visits was obtained from the Canadian Institute for Health Information’s Discharge Abstract Database and the National Ambulatory Care Reporting System database. We obtained additional covariate information from the Ontario Health Insurance Plan database, which includes health claims for inpatient and outpatient physician services. A subpopulation of patients had outpatient hemoglobin A1c (HbA1c) values available in the 1 year prior to the relevant study quarter.

International Classification of Diseases 9^th^ revision (ICD-9, pre-2002), 10^th^ Revision (ICD-10, post-2002), Canadian Classification of Diagnostic, Therapeutic, and Surgical Procedures (CCP, pre-2002) and Canadian Classification of Health Interventions (CCI, post-2002) codes were used to assess baseline comorbidities in the 5 years prior to 3 study quarters (administrative codes listed in S2 Table). Codes utilized to ascertain hypoglycemia encounters are detailed in S3 Table.

### Patients

During each quarter, we identified all adults with diabetes as defined by the ODD. We then excluded the following patients from analysis: 1) those with a missing age or sex, invalid age (over 105 years) or death recorded on or before the beginning of the quarter (for data cleaning purposes), 2) non-Ontarian residents at the beginning of each quarter (to allow for adequate patient follow-up), and 3) those under the age of 66 (as the province’s drug formulary provides prescription coverage to those over the age of 65 and to avoid incomplete medication records in their first year of eligibility).

We defined patients with treated diabetes as those who had evidence of at least 1 antihyperglycemic prescription (including insulin or an oral antihyperglycemic medication) during the study quarter, insulin users as those with evidence of at least 1 prescription for insulin during the study quarter, and patients with newly treated diabetes as those who had evidence of at least 1 antihyperglycemic medication prescription during the quarter with no evidence of a previous prescription for any other antihyperglycemic medication (any insulin or oral agent) in the 1 year prior. Monotherapy users had evidence of only 1 antihyperglycemic medication prescription during the relevant quarter and combination therapy users had evidence of more than 1 prescription.

### Outcomes

For the primary outcome, we examined the percentage of treated and newly treated patients with a prescription for insulin, sulphonylureas, alpha-glucosidase inhibitors, metformin, thiazolidinediones, meglitinides, and dipeptidyl peptidase-4 inhibitors (DPP-4). These antihyperglycemic medications are the only agents currently covered by our provincial drug formulary. For our secondary outcome we examined the percentage of treated patients with a hospital encounter with hypoglycemia (emergency room visit or inpatient admission) during each quarter of study.

### Statistical Analysis

We used descriptive statistics to summarize the baseline characteristics of patients with treated and newly treated diabetes at the beginning of three study quarters (April 1 2002, April 1, 2007, April 1, 2012) (age, sex, income quintile, residential status, presence of chronic kidney disease, chronic liver disease, cancer, coronary artery disease, congestive heart failure, peripheral vascular disease, dementia, stroke/transient ischemic attack, diabetic neuropathy, retinopathy, number of laboratory tests, eye exams and HbA1c values). For each characteristic, we compared differences across the study quarters using Chi-squared tests for categorical variables and Kruskal-Wallis tests for continuous variables.

The percentage of patients prescribed each antihyperglycemic medication during the relevant quarter was calculated by dividing the total number with a prescription (numerator) by the total number of treated patients (or newly treated patients) (denominator) during the quarter. The percentage of patients with a hypoglycemia encounter during each quarter was determined by dividing the total number of patients with at least 1 encounter (numerator) by the total number of treated patients (denominator). We conducted all analyses with SAS version 9.3 (SAS Institute, Cary, North Carolina).

## Results

**Fig 1:**
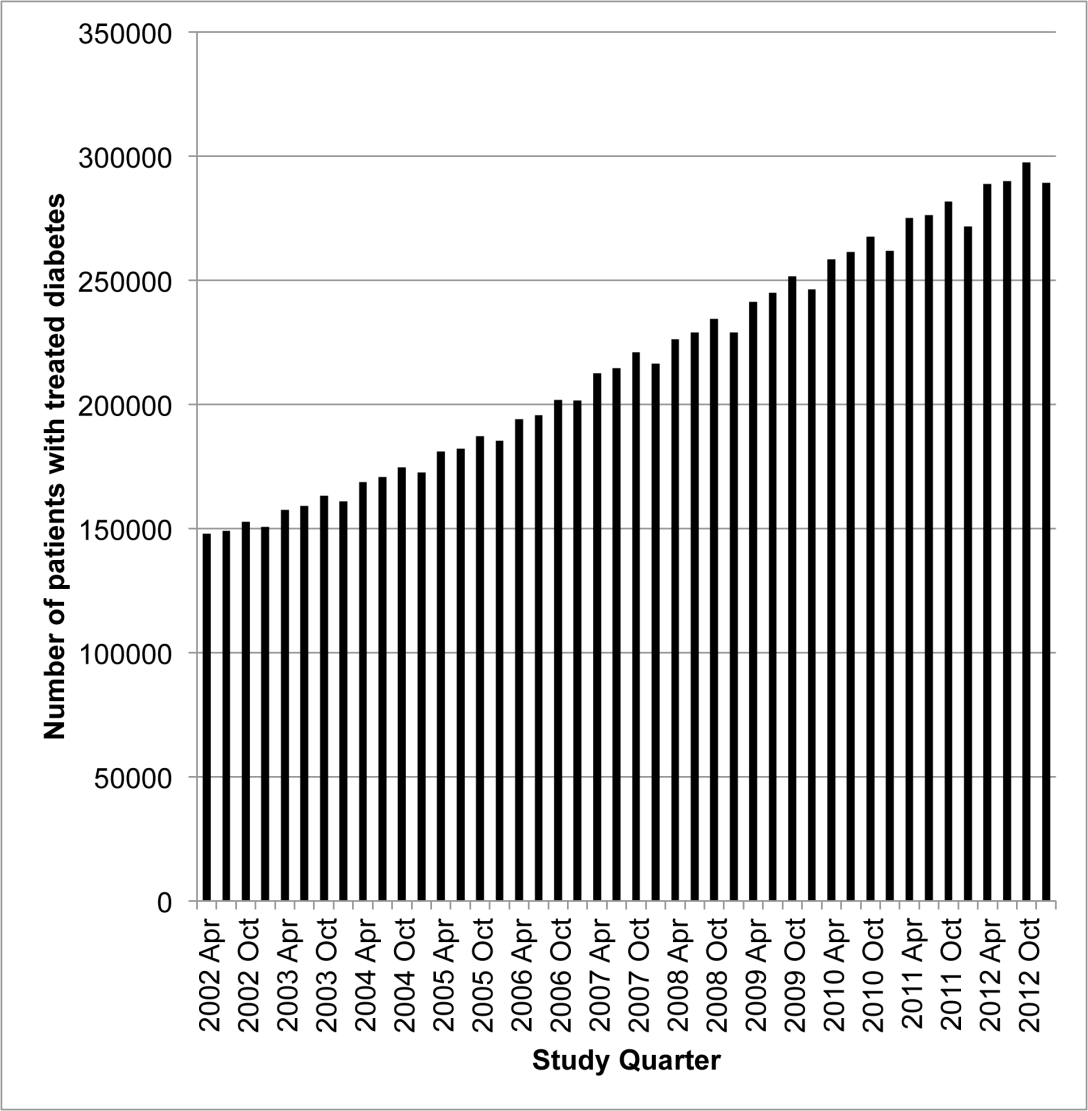
The number of patients with treated diabetes has nearly doubled over the last decade (2002–2013).

Over the decade from April 2002 until March 2013, the number of patients with treated diabetes almost doubled from 148,021 to 289,312 individuals (Fig 1). The baseline characteristics of treated patients are presented in Table 1. Their mean age appeared to increase slightly over the study quarters and the proportion that were women appeared to decline. The percentage of patients with chronic kidney disease, cancer, dementia and neuropathy increased over the study period while the percentage with chronic liver disease, coronary artery disease, congestive heart failure, peripheral vascular disease, stroke or transient ischemic attack, retinopathy, and a major eye exam in the year previous appeared to decline. Further, their mean number of cholesterol, HbA1c, and creatinine tests increased while their number of glucose tests decreased. Where available for a sub-population of included patients, HbA1c values appeared to increase slightly. The demographic characteristics, comorbidities and health care utilization of patients with newly treated diabetes are illustrated in S4 Table.

**Table 1:** Baseline characteristics of patients with treated diabetes.

	April 1, 2002		April 1, 2007		April 1, 2012		P Values
	N = 148,021	%	N = 212,538	%	N = 288,866	%	
Age (yrs)							
Mean (SD)	74.7 (6.3)		75.1 (6.5)		75.4 (6.8)		< .0001
Median (IQR)	74 (70–79)		74(70–80)		74 (70–80)		
66–69	35,472	24.0%	49,710	23.4%	69,073	23.9%	
70–74	44,063	29.8%	59,111	27.8%	76,954	26.6%	
75–79	35,821	24.2%	50,384	23.7%	63,877	22.1%	
80–84	20,465	13.8%	33,387	15.7%	45,993	15.9%	
85–89	9105	6.2%	14 839	7.0%	24,064	8.3%	
90+	3095	2.1%	5107	2.4%	8905	3.1%	
Sex—Female	76,456	51.7%	107,187	50.4%	140,884	48.8%	< .0001
Income quintile							
1 (lowest)	35,308	23.9%	49,607	23.3%	62,975	21.8%	< .0001
2	34,709	23.5%	47,862	22.5%	63,610	22.0%	
3	29,639	20.0%	41,770	19.7%	57,919	20.1%	
4	25,418	17.2%	38,819	18.3%	55,395	19.2%	
5 (highest)	22,482	15.2%	33,671	15.8%	47,779	16.5%	
Missing	465	0.3%	809	0.4%	1188	0.4%	
Rural location							
No	125,609	84.9%	183,482	86.3%	250,090	86.6%	< .0001
Yes	22,340	15.1%	28,984	13.6%	38,654	13.4%	
Missing	72	0.1%	72	0.03%	122	0.04%	
Comorbidities^a^							
Chronic kidney disease	15,277	10.3%	24,665	11.6%	41,473	14.4%	< .0001
Chronic liver disease	5650	3.8%	7963	3.8%	10,577	3.7%	0.03
Any cancer	37,955	25.6%	55,425	26.1%	79,749	27.6%	< .0001
Coronary artery disease	55,221	37.3%	73,074	34.4%	86,904	30.1%	< .0001
Congestive heart failure	30,419	20.6%	36,450	17.2%	43,059	14.9%	< .0001
Peripheral vascular disease	6000	4.1%	5666	2.7%	4706	1.6%	< .0001
Dementia	14,096	9.5%	23,644	11.1%	35,577	12.3%	< .0001
Stroke/TIA	8182	5.5%	8478	4.0%	9329	3.2%	< .0001
Neuropathy	1640	1.1%	2683	1.3%	4085	1.4%	< .0001
Retinopathy	5172	3.5%	4964	2.3%	4563	1.6%	< .0001
Investigations^b^							
Mean (SD) number cholesterol tests	1.1 (1.3)	—-	1.3 (1.3)	—-	1.4 (1.2)	—-	< .0001
Median (IQR) cholesterol tests	1 (0–2)	—-	1 (0–2)	—-	1 (1–2)	—-	
Mean (SD) HbA1c tests	1.9 (1.9)	—-	2.0 (1.7)	—-	2.2 (1.6)	—-	< .0001
Median (IQR) HbA1c tests	2 (0–3)	—-	2 (1–3)	—-	2 (1–3)	—-	
Mean (SD) creatinine tests	1.9 (2.3)	—-	2.2 (2.3)	—-	2.4 (2.3)	—-	< .0001
Median (IQR) creatinine tests	1 (0–3)	—-	2 (1–3)	—-	2 (1–3)	—-	
Mean (SD) glucose tests	2.7 (3.3)	—-	2.3 (2.5)	—-	2.2 (2.0)	—-	< .0001
Median (IQR) glucose tests	2 (1–4)	—-	2 (1–3)	—-	2 (1–3)	—-	
At least 1 eye exam	61,157	41.3%	81,240	38.2%	97,025	33.6%	< .0001
Laboratory Data^c^							
At least 1 HbA1c outpatient lab value	—-	—-	53,239	25.1%	75,311	26.1%	< .0001
Mean (SD HbA1c (%)	—-	—-	7.0% (1.2%)	—-	7.2% (1.2%)	—-	< .0001
Mean (SD) HbA1c (mmol/mol)			53 (13.1)		55 (13.1)		
Median (IQR) HbA1c	—-	—-	6.8% (6.2%-7.5%)	—-	7.0% (6.5%-7.7%)	—-	< .0001
Mean (SD) HbA1c (mmol/mol)			51 (44–58)		53 (48–61)		

Abbreviations: TIA transient ischemic attack, SD standard deviation, IQR interquartile range, HbA1c hemoglobin A1c

^a^Comorbidities were examined in the 5 years prior.

^b^Investigations were examined in the 1 year prior.

^c^For a sub-population, lab values were available in the 1 year prior.

### Patients with treated diabetes

**Fig 2:**
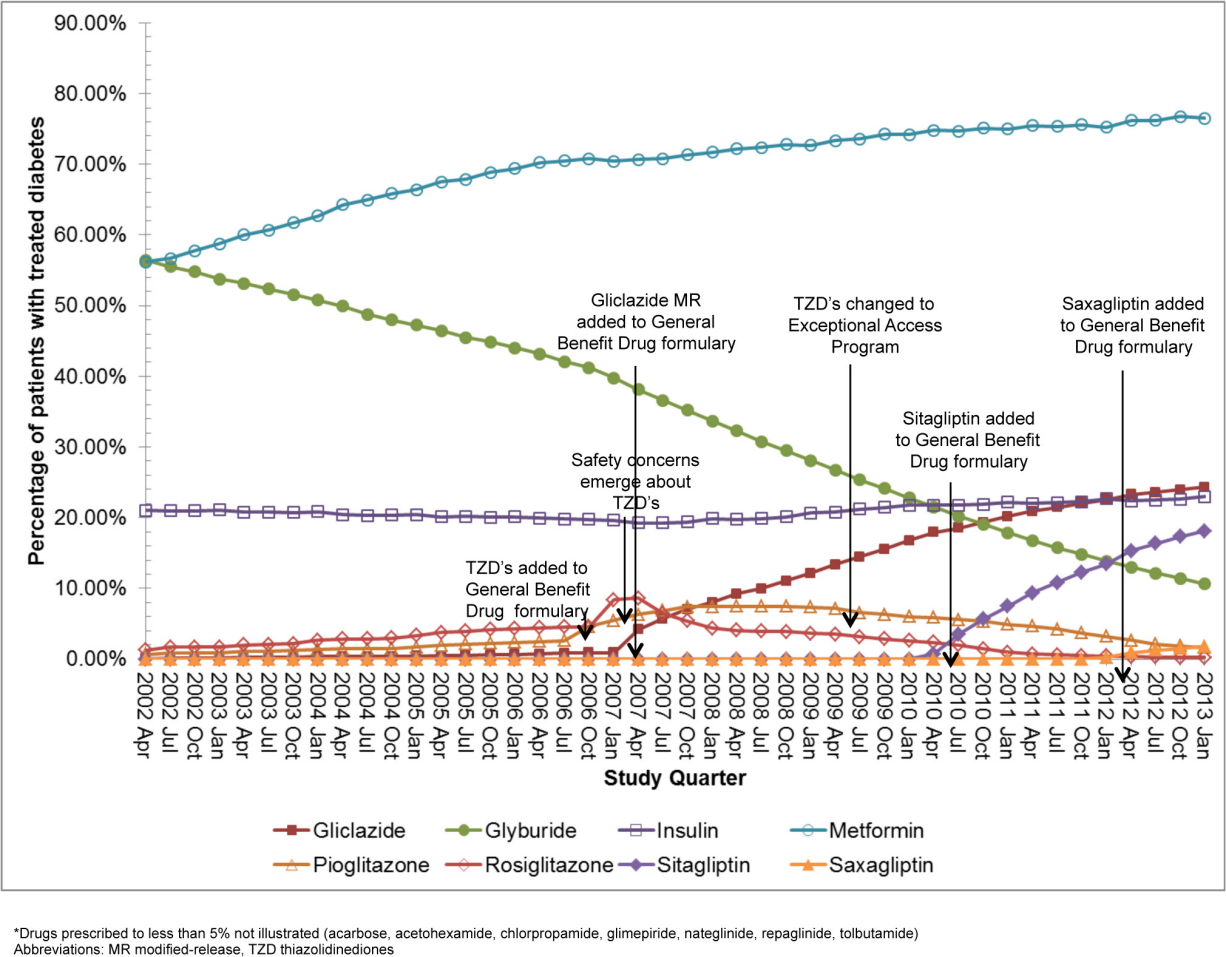
Antihyperglycemic medication prescriptions 2002–2013. The percentage prescribed metformin increased over the study period (56.2% in first quarter, 76.5% in last quarter), as did prescriptions for the DPP-4 inhibitors saxagliptin (prescriptions increased from 0% to 1.8% following its formulary introduction in 2012) and sitagliptin (prescriptions increased from 0% to 18.1% following its formulary introduction in 2010). A decline in glyburide prescriptions was evident (56.4% in the first quarter, 10.7% in the last quarter), while gliclazide prescriptions increased (prescriptions increased from 0.4% to 24.3% following the formulary introduction of modified-release gliclazide in 2007). Over the last 10 years about 20% of treated patients have been prescribed insulin. Further, after an initial increase following their introduction to the provincial formulary in 2006/2007, thiazolidinedione prescriptions declined, although pioglitazone less steeply. Prescriptions for acarbose, acetohexamide, glimepiride, repaglinide, tolbutamide, nateglinide, and chlorpropamide have remained low (less than 5% of patients had evidence of a prescription during each study quarter).

Fig 2 shows the percentage of patients with treated diabetes with a prescription for insulin, sulphonylureas, alpha-glucosidase inhibitors, metformin, meglitinides, thiazolidinediones and DPP-4 inhibitors from 2002 until 2013.

Antihyperglycemic mono and combination therapy is illustrated in S1 and S2 Figs. Over the last decade, there was a small decrease in the percentage of patients prescribed monotherapy (including insulin monotherapy), and a small increase in those prescribed 3 or more agents (including in insulin users). The oral antihyperglycemic medications prescribed in insulin users are illustrated in S3 Fig.

### Patients with newly treated diabetes

New antihyperglycemic medication prescriptions are illustrated in S4 Fig. The majority of patients were prescribed metformin (approximately 80%), with a small percentage decrease noted from July 2006 until April 2008. The percentage of patients prescribed the DPP-4 inhibitors increased (prescriptions for sitagliptin increased from 0% to 10.1% following its introduction to the formulary; saxagliptin prescriptions increased from 0% to 2.1% following its introduction to the formulary). We also note that fewer of these patients were prescribed glyburide over time, (39.0% in the first quarter and 2.9% in the last quarter) with an increasing number prescribed gliclazide (prescriptions increased from 0.3% to 11.7% following the introduction of modified-release gliclazide to the formulary). Insulin prescriptions remained relatively stable (approximately 7%). Further, although thiazolidinedione prescriptions initially rose in 2006/2007, they have since decreased. Prescriptions for acarbose, acetohexamide, glimepiride, repaglinide, tolbutamide, nateglinide, and chlorpropamide remained low (less than 5% of patients had evidence of a prescription during each study quarter).

Where mono- and combination therapy was examined in newly treated patients, there was a slight decrease in monotherapy prescriptions (including insulin monotherapy) and an increase in combination prescriptions over time (including insulin combination therapy) (S5 and S6 Figs).

### Hypoglycemia

**Fig 3:**
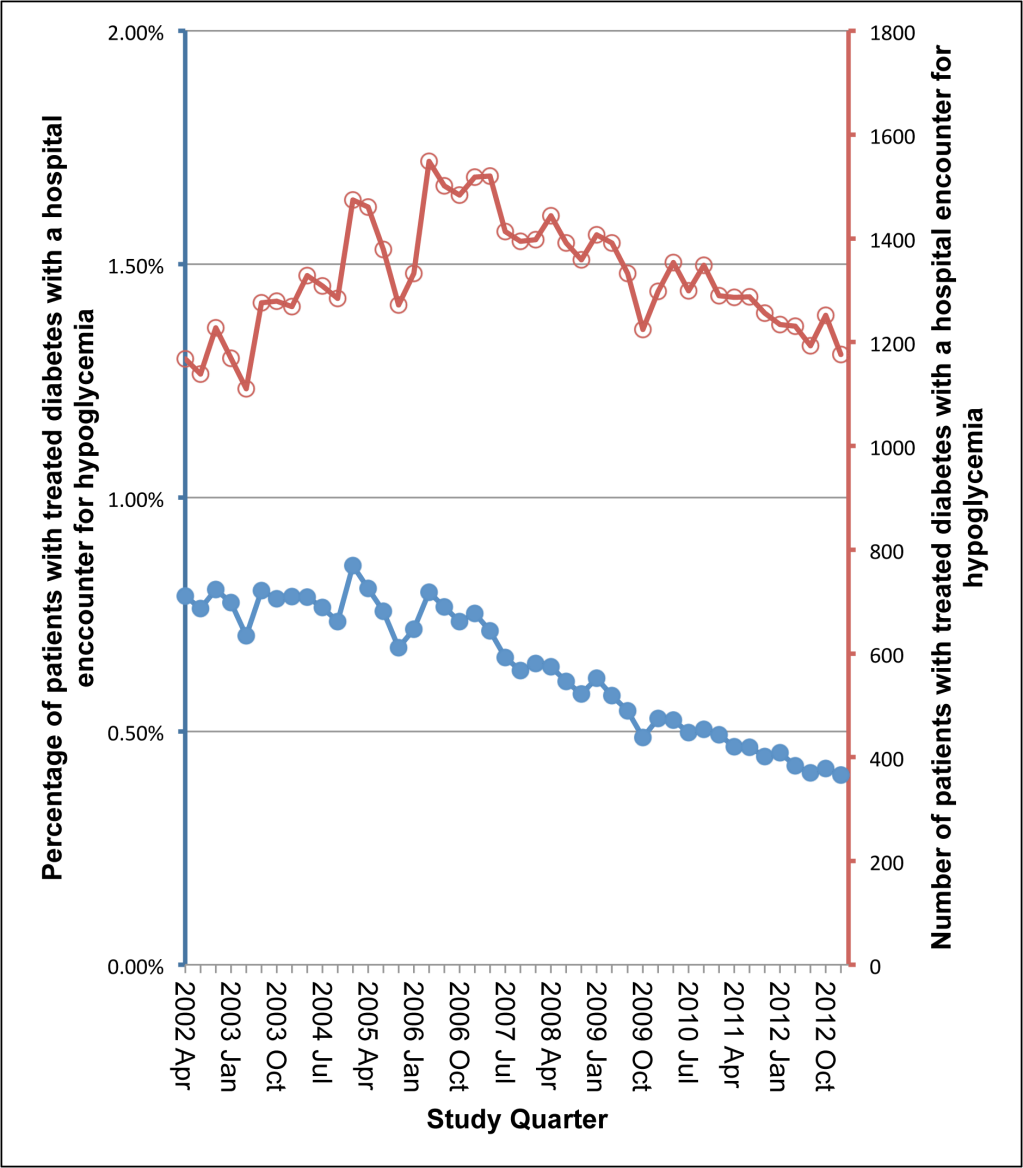
Hospital encounters for hypoglycemia in treated patients 2002–2013.

In the setting of these prescription trends, the absolute number of treated patients with a hypoglycemia encounter increased until mid-2006 and then declined. However, when the increasing prevalence of treated diabetes was accounted for, the percentage with a hospital encounter with hypoglycemia declined by 50% over the decade (0.8% with an event in the first quarter, 0.4% with an event in the last quarter). (Fig 3) This finding appeared to be consistent among age categories of older adults (66–74, 75–79, 80+ years) (S7 Fig)

## Discussion

In this study we have identified several trends in antihyperglycemic medication prescriptions in patients with diabetes age 66 and older in Ontario, Canada.

First, over the last decade there has been a substantial increase in the number of older adults prescribed antihyperglycemic medications in our province. Whether this increase is due to an increased detection of diabetes, an aging population, or a higher number of individuals with obesity and sedentary lifestyle remains to be determined.

Second, consistent with guidelines which recommend metformin as a first line agent for its efficacy, safety, weight effects, and possible cardiovascular benefit, [12,13] metformin remains the most commonly prescribed antihyperglycemic medication among older adults in Ontario. This result is consistent with high rates of metformin use in other jurisdictions. [14–18]

Third, we found that prescriptions for glyburide steadily declined over the last decade whereas those for gliclazide have increased. This change is consistent with clinical practice guidelines which have endorsed avoiding glyburide in older patients in favour of sulphonylureas including gliclazide that have a lower risk for hypoglycemia. [19]

Fourth, since their addition to the drug formulary, prescriptions for both pioglitazone and rosiglitazone have declined. These findings may reflect safety concerns that have arisen with these medications, [20–23] regulatory advisories (S5 Table), and funding status changes in our province (thiazolidinediones transferred from the unrestricted formulary to the Exceptional Access Program in in June 2009). [24,25] Pioglitazone currently remains more commonly prescribed than rosiglitazone perhaps reflecting evidence of its better safety profile compared with its counterpart. [26–28] Consistent with the findings of research in other regions, we also note that there has been more prescriptions for new medications including the DPP-4 inhibitors. [14,17,18]

Fifth, we found that that prescriptions for combinations of antihyperglycemic medications has increased over time, including in newly treated patients. It is possible that clinical trials that have suggested the benefit of intensive glycemic control in the prevention of microvascular complications have been contributory, [5,6] along with the possibility of personalizing therapy with several drugs in order to achieve better control. [16] Further, published reports have noted that combination therapy at submaximal doses may help to improve glycemic control more rapidly and with fewer side effects than monotherapy, [13,29–31] and practice guidelines suggest that combination therapy be initiated in patients with higher HbA1c’s. [13]

Finally, in the setting of these prescription trends, the overall percentage of treated patients with a hospital encounter for hypoglycemia has declined in our region. Our findings are consistent with a recent study of United States Medicare beneficiaries (1999 to 2011). When the changing prevalence of diabetes was accounted for by the authors, admissions for hypoglycemia decreased by 9.5%. [32] Although a decline in the use of glyburide and the uptake of agents associated with a lower hypoglycemic risk may have contributed to this trend, other factors including changes in the accuracy of diagnostic coding, diabetes screening, quality of patient care and education, [33] secular trends in glycemic control, and the characteristics of patients with the disease (comorbidities, functional limitations, self-management behavior), may have also played a role. [32,34]

### Strengths and Limitations

Compared with previous drug trend studies, our report has several strengths. [18,24,25,27] First, we comprehensively examined prescriptions for all 15 antihyperglycemic medications currently covered by the provincial drug formulary and ascertained prescription trends in a variety of antihyperglycemic medication users (including those with treated and newly treated diabetes). Our decade of study also allowed for an assessment of prescription trends during an era of changing diabetes care. Where previous studies have been limited to younger patients with diabetes, ours provided a perspective on prescribing practices in a more vulnerable population of older adults. We also detailed the demographic characteristics, comorbidities, and HbA1c values of included patients to help put prescribing practices into context. Finally, in the setting of changing prescription trends, we quantified both inpatient and emergency room hospital encounters with hypoglycemia–a serious adverse event in the elderly.

Our study has limitations. We were unable to capture antihyperglycemic medication prescriptions not covered by our provincial formulary (including glucagon-like peptide 1 agonists and sodium glucose co-transporter 2 inhibitors). Although we expect our results to be generalizable to the elderly with publically funded healthcare, we cannot extend our results to those under the age of 65 or on other drug funding schemes where variations in drug prescribing has been noted.

Our databases also did not allow us to evaluate diabetes type, although given their age and the prevalence of type 2 diabetes, the majority of patients likely had type 2 diabetes. Further, we could not capture their duration of diabetes which can influence treatment choices and diabetes-related complications. [34]

For our outcome of hypoglycemia, we were unable to assess events experienced outside of the hospital, including emergency medical service contacts. We were also unable to capture home events including those that were asymptomatic (as often the case in older adults whose symptoms are masked by medications including beta blockers or who have hypoglycemia unawareness), or those events that did not lead to hospital presentation. Additionally, we assessed the outcome of hypoglycemia with administrative codes which have limited sensitivity when compared to laboratory plasma glucose measurements (although the latter is not the best reference standard as treatment with glucose may have been initiated by the time plasma glucose is measured). Further, although we do note a decline in the use of glyburide and the uptake of safer medications, these data do not prove that prescription changes led to a decline in the rates of hypoglycemia. Although we did measure comorbidities and demographic characteristics that are associated with hypoglycemia, we were also unable to account for changes in health literacy, attitudes, and social support which could cause differences in the likelihood of seeking medical care. [33]

### Conclusions

Antihyperglycemic medication prescribing practices have changed significantly in Ontario over the last 11 years. In the setting of a decline in the use of glyburide, and the uptake of drugs with a lower hypoglycemia risk, there has been a decrease in the percentage of treated patients with a hospital encounter for hypoglycemia in our region. The extent to which this reduction is related to the use of safer medications or to other factors remains to be established.

## Supporting Information

S1 Fig Mono and combination therapy 2002–2013. (DOCX)

S2 Fig Insulin mono/combination therapy 2002–2013. (DOCX)

S3 Fig Oral antihyperglycemic medication prescriptions in insulin combination therapy users 2002–2013. (DOCX)

S4 Fig Antihyperglycemic medication prescriptions in patients with newly treated diabetes. (DOCX)

S5 Fig Mono/combination therapy in patients with newly treated diabetes 2002–2013. (DOCX)

S6 Fig Insulin mono/combination therapy in patients with newly treated diabetes 2002–2013. (DOCX)

S7 Fig Hospital encounters for hypoglycemia in treated patients by age category 2002–2013. (DOCX)

S1 Table Checklist of recommendations for reporting of observational studies using the STROBE guidelines. (DOCX)

S2 Table Coding definitions for demographic and comorbid conditions. (DOCX)

S3 Table Coding definitions for hospital presentation with hypoglycemia. (DOCX)

S4 Table Baseline characteristics of patients with newly treated diabetes. (DOCX)

S5 Table Timeline of safety events during study period. (DOCX)
