# Supplementary material for: Trends in Antihyperglycemic Medication Prescriptions and Hypoglycemia in Older Adults: 2002-2013
Source: PLoS One. 2015 Sep 3;10(9):e0137596. doi: 10.1371/journal.pone.0137596 (PMC4559313; doi:10.1371/journal.pone.0137596)
Supplement: S1 Fig — (DOCX) [file pone.0137596.s001.docx]

**S1 Fig. Mono and combination therapy 2002-2013**

*Most frequent 2 agent combinations over time were metformin and glyburide (2002-2010) and metformin and gliclazide (2011-2013).

Most frequent 3 agent combinations over time were metformin, glyburide and insulin (2002-2003), metformin, glyburide and rosiglitazone (2004-2006), metformin, glyburide and pioglitazone (2007-2010), and metformin, gliclazide and sitagliptin (2011-2013).
