# Supplementary material for: Trends in Antihyperglycemic Medication Prescriptions and Hypoglycemia in Older Adults: 2002-2013
Source: PLoS One. 2015 Sep 3;10(9):e0137596. doi: 10.1371/journal.pone.0137596 (PMC4559313; doi:10.1371/journal.pone.0137596)
Supplement: S4 Fig — (DOCX) [file pone.0137596.s004.docx]

**S4 Fig. Antihyperglycemic medication prescriptions in patients with newly treated diabetes 2002-2013**

*Drugs prescribed to less than 5% not illustrated (acarbose, acetohexamide, chlorpropamide, glimepiride, nateglinide, repaglinide, tolbutamide)
