# Supplementary material for: Trends in Antihyperglycemic Medication Prescriptions and Hypoglycemia in Older Adults: 2002-2013
Source: PLoS One. 2015 Sep 3;10(9):e0137596. doi: 10.1371/journal.pone.0137596 (PMC4559313; doi:10.1371/journal.pone.0137596)
Supplement: S7 Fig — (DOCX) [file pone.0137596.s007.docx]

**S7 Figure. Hospital encounters for hypoglycemia in treated patients by age category 2002-2013**
