# Supplementary material for: Trends in Antihyperglycemic Medication Prescriptions and Hypoglycemia in Older Adults: 2002-2013
Source: PLoS One. 2015 Sep 3;10(9):e0137596. doi: 10.1371/journal.pone.0137596 (PMC4559313; doi:10.1371/journal.pone.0137596)
Supplement: S2 Table — (DOCX) [file pone.0137596.s009.docx]

**S2 Table. Coding definitions for demographic and comorbid conditions**

| **Characteristics/**  **Condition** | **Database** | **Codes** |
| --- | --- | --- |
| Age | RPDB |  |
| Sex | RPDB |  |
| Income quintile | Statistics Canada |  |
| Rural location | Statistics Canada |  |
| Chronic kidney disease | CIHI-DAD  OHIP | ICD 9: "4030", "4031", "4039", "4040", "4041", "4049", "585", "586", "5888", "5889", "2504"  ICD 10: "E102", "E112", "E132", "E142", "I12", "I13", "N08", "N18", "N19"  OHIP DX: "403", "585" |
| Chronic liver disease | CIHI-DAD  OHIP | ICD 9: "4561", "4562", "070", "5722", "5723", "5724", "5728", "573", "7824", "V026", "2750", "2751", "7891", "7895", "571"  ICD 10: "B16", "B17", "B18", "B19", "I85", "R17", "R18", "R160", "R162", "B942", "Z225", "E831", "E830", "K70", "K713", "K714", "K715", "K717", "K721", "K729", "K73", "K74", "K753", "K754", "K758", "K759", "K76", "K77"  OHIP DX: "571", "573", "070"  OHIP FEE: "Z551", "Z554" |
| Any cancer | CIHI-DAD  OHIP | ICD 9: "V10", "140", "141", "142", "143", "144", "145", "146", "147", "148", "149", "150", "151", "152", "153", "154", "155", "156", "157", "158", "159", "160", "161", "162", "163", "164", "165", "170", "171", "172", "173", "174", "175", "176", "179", "180", "181", "182", "183", "184", "185", "186", "187", "188", "189", "190", "191", "192", "193", "194", "1950", "1951", "1952", "1953", "1954", "1955", "1958", "196", "197", "198", "1990", "1991", "2000", "2001", "2002", "2008", "2010", "2011", "2012", "2014", "2015", "2016", "2017", "2019", "2020", "2026", "2028", "2029", "203", "204", "205", "206", "207", "208", "230", "231", "232", "233", "234"  ICD 10: "80003", "80006", "80013", "80023", "80033", "80043", "80102", "80103", "80106", "80113", "80123", "80203", "80213", "83123", "87202", "87203", "959", "965", "966", "967", "968", "969", "970", "971", "980", "982", "984", "985", "986", "987", "988", "989", "990", "991", "993", "C00", "C01", "C02", "C03", "C04", "C05", "C06", "C07", "C08", "C09", "C10", "C11", "C12", "C13", "C14", "C15", "C16", "C17 C18", "C19", "C20", "C21", "C22", "C23", "C24", "C25", "C26", "C30", "C31", "C32", "C33", "C34", "C37", "C38", "C39", "C40", "C41", "C43", "C44", "C45”, “C46", "C47", "C48", "C49", "C50", "C51", "C52", "C53", "C54", "C55", "C56", "C57", "C58", "C60", "C61", "C62", "C63", "C64", "C65", "C66", "C67", "C68", "C69", "C70", "C71", "C72", "C73", "C74", "C75" ,"C76", "C77", "C78", "C79", "C80", "C81", "C82", "C83", "C84", "C85", "C90", "C91", "C92", "C93", "C94", "C95", "C96", "C97", "D00", "D01", "D02", "D03", "D04", "D05", "D06", "D07", "D09"  OHIP DX: "140", "141", "142", "143", "144", "145", "146", "147", "148", "149", "150", "151", "152", "153", "154", "155", "156", "157", "158", "159", "160", "161", "162", "163 164"," 165", "170", "171", "172", "173", "174", "175", "179", "180", "181", "182", "183", "184", "185", "186", "187", "188", "189 190", "191", "192", "193", "194", "195", "196", "197", "198", "199", "200", "201", "202", "203", "204", "205", "206", "207", "208" |
| Coronary artery disease (excluding angina) | CIHI-DAD  OHIP | ICD 9: "412", "410"  ICD 10: "I21", "I22", "Z955", "T822"  CCI: "1IJ50", "1IJ76"  CCP: "4801", "4802", "4803", "4804", "4805", "481", "482", "483"  OHIP FEE: "R741", "R742", "R743", "G298", "E646", "E651", "E652", "E654", "E655", "Z434", "Z448"  OHIP DX: "410", "412" |
| Congestive heart failure | CIHI-DAD  OHIP | ICD 9: "425", "5184", "514", "428"  ICD 10: "I500", "I501", "I509", "I255", "J81"  CCP: "4961", "4962", "4963", "4964"  CCI: "1HP53", "1HP55", "1HZ53GRFR", "1HZ53LAFR", "1HZ53SYFR"  OHIP FEE: "R701", "R702", "Z429"  OHIP DX: "428" |
| Peripheral vascular disease | CIHI-DAD  OHIP | ICD 9: "4402", "4408", "4409", "5571", "4439", "444"  ICD 10: "I700", "I702", "I708", "I709", "I731", "I738", "I739", "K551"  CCP: "5125", "5129", "5014", "5016", "5018", "5028", "5038"  CCI: "1KA76", "1KA50", "1KE76", "1KG26", "1KG50", "1KG57", "1KG76MI", "1KG87"  OHIP FEE: "R787", "R780", "R797", "R804", "R809", "R875", "R815", "R936", "R783", "R784","R785", "E626", "R814", "R786", "R937", "R860", "R861", "R855", "R856", "R933", "R934", "R791", "E672", "R794", "R813", "R867", "E649" |
| Dementia | CIHI-DAD  OHIP | ICD 9: "2900", "2901", "2903", "2904", "2908", "2909", "2948", "2949", "3310", "3311", "3312", "2941", "797"  ICD 10: "F065", "F066", "F068", "F069", "F09", "F00", "F01", "F02", "F03", "F051", "G30", "G31", "R54"  OHIP DX: "290","331", "797" |
| Stroke/  Transient ischemic attack | CIHI-DAD | ICD 9: "430", "431", "434", "435", "436"  ICD 10: "I630", "I631", "I632", "I633", "I634", "I635", "I638", "I639", "I64", "H341", "I600", "I601", "I602", "I603", "I604", "I605", "I606", "I607", "I609", "I61", "G450", "G451", "G452", "G453", "G458", "G459" |
| Diabetic neuropathy | CIHI-DAD | ICD 9: "3572"  ICD 10: “E1040”,”E10400”, “E10401”,”E10402”,“E10403”, “E10404”, “E10409”, “E1041”, “E10410”, “E10411”, “E10412”, “E10413”, “E10414”, “E10419”, “E1042”, “E10420”, “E10421”, “E10422”, “E10423”,“E10424”,“E10429”,“E10480”,“E10481”,“E10482”, “E10483”,“E10484”, “E10489”, “E10490”, “E10491”, “E10492”, “E10493”, “E10494”, “E10499”, “E1140”, “E11400”, “E11401”, “E11402”, “E11403”, “E11404”, “E11409”, “E1141”, “E11410”, “E11411”, “E11412”, “E11413”, “E11414”, “E11419”, “E1142”, “E11420”, “E11421”, “E11422”, “E11423”, “E11424”, “E11429”, “E11480”, “E1148”, “E11482”, “E11483”, “E11484”, “E11489”, “E11490”, “E11491”, “E11492”, “E11493”, “E11494”, “E11499”, “E1340”, “E13400”, “E13401”, “E13402”, “E13403”, “E13404”, “E13409”, “E1341”, “E13410”, “E13411”, “E13412”, “E13413”, “E13414”, “E13419”, “E1342”, “E13420”, “E13421”, “E13422”, “E13423”, “E13424”, “E13429”, “E13480”, “E13481”, “E13482”, “E13483”, “E13484”, “E13489”, “E13490”, “E13491”, “E13492”, “E13493”, “E13494”, “ “E13499”, “E1440”, “E14400”, “E14401”, “E14402”, “E14403”, “E14404”, “E14409”, “E1441”, “E14410”, “E14411”, “E14412”, “E14413”, “E14414”, “E14419”, “E1442”, “E14420”, “E14421”, “E14422”, “E14423”, “E14424”, “E14429”, “E14480”, “E14481”, “E14482”, “E14483”, “E14484”, “E14489”, “E14490”, “E14491”, “E14492”, “E14493”, “E14494”, “E14499”, “G590”, “G632” |
| Retinopathy | CIHI-DAD | ICD 9: “36201”, “36202”, “36210”, “36212”, “36229”    ICD 10: “E1030”, “E10300”, “E10301”, “E10302”, “E10303”, “E10304”, “E10309”, “E1031”, “E10310”, “E10311”, “E10312”, “E10313”, “E10314”, “E10319”, “E1032”, “E10320”, “E10321”, “E10322”, “E10323”, “E10324”, “E10329”, “E1033”, “E10330”, “E10331”, “E10332”, “E10333”, “E10334”, “E10339”, “E10340”, “E10341”, “E10342”, “E10343”, “E10344”, “E10349”, “E1130”, “E11300”, “E11301”, “E11302”, “E11303”, “E11304”, “E11309”, “E1131”, “E11310”, “E11311”, “E11312”, “E11313”, “E11314”, “E11319”, “E1132”, “E11320”, “E11321”, “E11322”, “E11323”, “E11324”, “E11329”, “E1133”, “E11330”, “E11331”, “E11332”, “E11333”, “E11334”, “E11339”, “E11340”, “E11341”, “E11342”, “E11343”, “E11344”, “E11349”, “H360” |
| Number of cholesterol tests | OHIP | OHIP FEE: “L055” |
| Number of HbA1c tests | OHIP | OHIP FEE: “L093" |
| Number of creatinine tests | OHIP | OHIP FEE: OHIP FEE: "L065", "L067", "L068" |
| Number of glucose tests | OHIP | OHIP FEE: "L111" |
| Major eye examination | OHIP | OHIP FEE: "A112", "A233", "A234", "A235", "A236", A239", "V401", "V406", "V402" |

Abbreviations: CIHI-DAD, Canadian Institute for Health Information’s Discharge Abstract Database; CCI, Canadian Classification of Health Interventions; CCP, Canadian Classification of Diagnostic, Therapeutic, and Surgical Procedures; HbA1c, hemoglobin A1c; ICD 9, International Classification of Diseases 9^th^ Revision; ICD 10, International Classification of Diseases 10^th^ Revision; OHIP, Ontario Health Insurance Plan; RPDB, Registered Persons Database of Ontario
