# Supplementary material for: Trends in Antihyperglycemic Medication Prescriptions and Hypoglycemia in Older Adults: 2002-2013
Source: PLoS One. 2015 Sep 3;10(9):e0137596. doi: 10.1371/journal.pone.0137596 (PMC4559313; doi:10.1371/journal.pone.0137596)
Supplement: S3 Table — (DOCX) [file pone.0137596.s010.docx]

**S3 Table. Coding definitions for hospital presentation with hypoglycemia**

| **Condition** | **Database** | **Codes** |
| --- | --- | --- |
| Hypoglycemia | CIHI-DAD  NACRS | ICD 10: E15, E160, E161, E162, E1063, E1163, E1363, E1463 |

Abbreviations: CIHI-DAD, Canadian Institute for Health Information Discharge Abstract Database; ICD 10, International Classification of Diseases 10^th^ Revision; NACRS, National Ambulatory Care Reporting System Database
