# Supplementary material for: Trends in Antihyperglycemic Medication Prescriptions and Hypoglycemia in Older Adults: 2002-2013
Source: PLoS One. 2015 Sep 3;10(9):e0137596. doi: 10.1371/journal.pone.0137596 (PMC4559313; doi:10.1371/journal.pone.0137596)
Supplement: S4 Table — (DOCX) [file pone.0137596.s011.docx]

**S4 Table. Baseline characteristics of patients with newly treated diabetes**

|  | April 1, 2002 | | April 1, 2007 | | April 1, 2012 | | P values |
| --- | --- | --- | --- | --- | --- | --- | --- |
|  | N=3498 | % | N=5863 | % | N=4478 | % |  |
| Age (yr) |  |  |  |  |  |  |  |
| Mean (SD) | 74.2 (6.2) |  | 74.5 (6.43) |  | 74.3 (6.63) |  | 0.1417 |
| Median (IQR) | 73 (69-78) |  | 73 (69-78) |  | 73 (69-79) |  |  |
| 66-69 | 926 | 26.5% | 1599 | 27.3% | 1314 | 29.3% |  |
| 70-74 | 1083 | 31.0% | 1661 | 28.3% | 1275 | 28.5% |  |
| 75-79 | 801 | 22.9% | 1337 | 22.8% | 911 | 20.3% |  |
| 80-84 | 416 | 11.9% | 780 | 13.3% | 570 | 12.7% |  |
| 85-89 | 200 | 5.7% | 355 | 6.1% | 297 | 6.6% |  |
| 90+ | 72 | 2.1% | 131 | 2.2% | 111 | 2.5% |  |
| Sex - Female | 1686 | 48.2% | 2765 | 47.2% | 2125 | 47.5% | 0.6192 |
| Income quintile |  |  |  |  |  |  |  |
| 1 (lowest) | 797 | 22.8% | 1 238 | 21.1% | 882 | 19.7% | <.0001 |
| 2 | 834 | 23.8% | 1 261 | 21.5% | 947 | 21.2% |  |
| 3 | 679 | 19.4% | 1 110 | 18.9% | 923 | 20.6% |  |
| 4 | 625 | 17.9% | 1 118 | 19.1% | 901 | 20.1% |  |
| 5 (highest) | 554 | 15.8% | 1 112 | 19.0% | 817 | 18.2% |  |
| Missing | 9 | 0.3% | 24 | 0.4% | 8 | 0.2% |  |
| Rural location |  |  |  |  |  |  |  |
| No | 2955 | 84.5% | 5146 | 87.8% | 3890 | 86.9% | <.0001 |
| Yes | 542 | 15.5% | 715 | 12.2% | 587 | 13.1% |  |
| Missing | ≤5 | --- | ≤5 | --- | ≤5 | --- |  |
| Comorbidities^a^ |  |  |  |  |  |  |  |
| Chronic kidney  disease | 167 | 4.8% | 575 | 9.8% | 396 | 8.8% | <.0001 |
| Chronic liver  disease | 130 | 3.7% | 236 | 4.0% | 170 | 3.8% | 0.7167 |
| Any cancer | 920 | 26.3% | 1508 | 25.7% | 1153 | 25.8% | 0.8021 |
| Coronary artery  disease | 1136 | 32.5% | 1832 | 31.3% | 1202 | 26.8% | <.0001 |
| Congestive heart  failure | 559 | 16.0% | 800 | 13.6% | 513 | 11.5% | <.0001 |
| Peripheral  vascular disease | 100 | 2.9% | 129 | 2.2% | 55 | 1.2% | <.0001 |
| Dementia | 271 | 7.8% | 488 | 8.3% | 444 | 9.9% | 0.0012 |
| Stroke/TIA | 139 | 4.0% | 185 | 3.2% | 135 | 3.0% | 0.0396 |
| Diabetic  neuropathy | ≤5 | --- | 21 | 0.4% | 29 | 0.7% | 0.0015 |
| Retinopathy | 13 | 0.4% | 44 | 0.8% | 17 | 0.4% | 0.0116 |
| Investigations^b^ |  |  |  |  |  |  |  |
| Mean (SD)  number  cholesterol tests | 1.0 (1.2) | --- | 1.2 (1.2) | --- | 1.1 (1.1) | --- | <.0001 |
| Median (IQR)  cholesterol tests | 1 (0-1) | --- | 1 (0-2) | --- | 1 (0-2) | --- |  |
| Mean (SD)  HbA1c tests | 1.3 (1.5) | --- | 1.5 (1.6) | --- | 1.5 (1.35) | --- | <.0001 |
| Median (IQR)  HbA1c tests | 1 (0-2) | --- | 1 (0-2) | --- | 1 (0-2) | --- |  |
| Mean (SD)  creatinine tests | 1.5 (1.9) | --- | 1.8 (2.1) | --- | 1.8 (1.91) | --- | <.0001 |
| Median (IQR)  creatinine tests | 1 (0-2) | --- | 1 (1-2) | --- | 1 (1-2) | --- |  |
| Mean (SD)  glucose tests | 2.0 (2.4) | --- | 1.9 (2.0) | --- | 1.6 (1.59) | --- | <.0001 |
| Median (IQR)  glucose tests | 1 (0-3) | --- | 2 (1-3) | --- | 1 (0-2) | --- |  |
| At least 1 eye  exam | 1 113 | 31.8% | 1 887 | 32.18% | 1,127 | 25.2% | <.0001 |
| Laboratory Data^c^ |  |  |  |  |  |  |  |
| At least 1 HbA1c  outpatient lab  value | --- | --- | 1 235 | 21.06% | 994 | 22.2% | 0.165 |
| Mean (SD)  HbA1c (%) | --- | --- | 6.8% (1.1%) | --- | 7.2% (1.2%) | --- | <.0001 |
| Mean (SD)  HbA1c  (mmol/mol) |  |  | 51 (12) |  | 55 (13.1) |  |  |
| Median (IQR)  HbA1c | --- | --- | 6.6% (6.1%-7.2%) | --- | 6.9% (6.5%-7.5%) | --- | <.0001 |
| Median (IQR)  HbA1c  (mmol/mol) |  |  | 49 (43-55) |  | 52 (48-58) |  |  |

Abbreviations: TIA transient ischemic attack, SD standard deviation, IQR interquartile range, HbA1c hemoglobin A1c

For privacy, cell sizes less than 6 were suppressed.

^a^Comorbidities were examined in the 5 years prior.

^b^Investigations were examined in the 1 year prior.

^c^For a subpopulation, lab values were available in the 1 year prior.
