# Supplementary material for: Trends in Antihyperglycemic Medication Prescriptions and Hypoglycemia in Older Adults: 2002-2013
Source: PLoS One. 2015 Sep 3;10(9):e0137596. doi: 10.1371/journal.pone.0137596 (PMC4559313; doi:10.1371/journal.pone.0137596)
Supplement: S5 Table — (DOCX) [file pone.0137596.s012.docx]

**S5 Table. Timeline of safety events during the study period– thiazolidinediones**

| October 2006 | Pioglitazone added to the province’s general benefit drug formulary |
| --- | --- |
| January 2007 | Rosiglitazone added to the province’s general benefit drug formulary |
| February 2007 | Safety signals emerge re: fracture risk with rosiglitazone [1] |
| May 2007 | Regulatory warnings re: cardiac safety of rosiglitazone [2,3] |
| June 2007 | Meta-analysis on cardiac safety of rosiglitazone published in the New England Journal of Medicine [4] |
| Nov 2007 | Black box warning issued for rosiglitazone in the United States [5] |
| June 2009 | Funding status for thiazolidinediones changed from General Benefit to the Exceptional Access Program in Ontario [6] |
| Sept 2010 | Prescribing restrictions on thiazolidinediones placed in the United States [7] |
| June 2011 | Regulatory attention to risk of bladder cancer with pioglitazone therapy [8] |

**1.** Health Canada. Important safety information on rosiglitazone-containing products: AVANDIA®, AVANDAMET® and AVANDARYL™ [Internet]**. 2007. Available:** http://www.healthycanadians.gc.ca/recall-alert-rappel-avis/hc-sc/2007/13994a-eng.php.

**2. Health Canada.** Cardiac Safety of Avandia (rosiglitazone maleate) - For Health Professional [Internet]. 2007.Available: http://www.healthycanadians.gc.ca/recall-alert-rappel-avis/hc-sc/2007/14440a-eng.php.

3.US Food and Drug Administration. Information for Healthcare Professionals Rosiglitazone maleate (marketed as Avandia, Avandamet, and Avandaryl) [Internet]. 2007. Available: http://www.fda.gov/Drugs/DrugSafety/ PostmarketDrugSafetyInformationforPatientsandProviders/ucm143460.htm..

4. Nissen SE, Wolski K. Effect of rosiglitazone on the risk of myocardial infarction and death from cardiovascular causes. N Engl J Med 2007; 356: 2457-71.

5.US Food and Drug Administration. FDA Adds Boxed Warning for Heart-related Risks to Anti-diabetes Drug Avandia [Internet]. 2007. Available: http://www.fda.gov/NewsEvents/Newsroom/ PressAnnouncements/2007/ucm109026.htm.

6.Ontario Ministry of Health and Long-term Care. Change in Funding Status Rosiglitazone and Pioglitazone [Internet]. 2009. Available: http://www.health.gov.on.ca/en/pro/programs/drugs/opdp_eo/notices/notices_docs/tzd_faq.pdf.

7.US Food and drug Administration. FDA significantly restricts access to the diabetes drug Avandia [Internet]. 2010. Available: http://www.fda.gov/ Drugs/DrugSafety/PostmarketDrugSafetyInformationforPatientsandProviders/ucm226956.htm.

8. Health Canada. Health Canada reviewing diabetes drug pioglitazone (Actos) and potential risk of bladder cancer [Internet]. 2011. Available: http://www.healthycanadians.gc.ca/recall-alert-rappel-avis/hc-sc/2011/13617a-eng.php.
